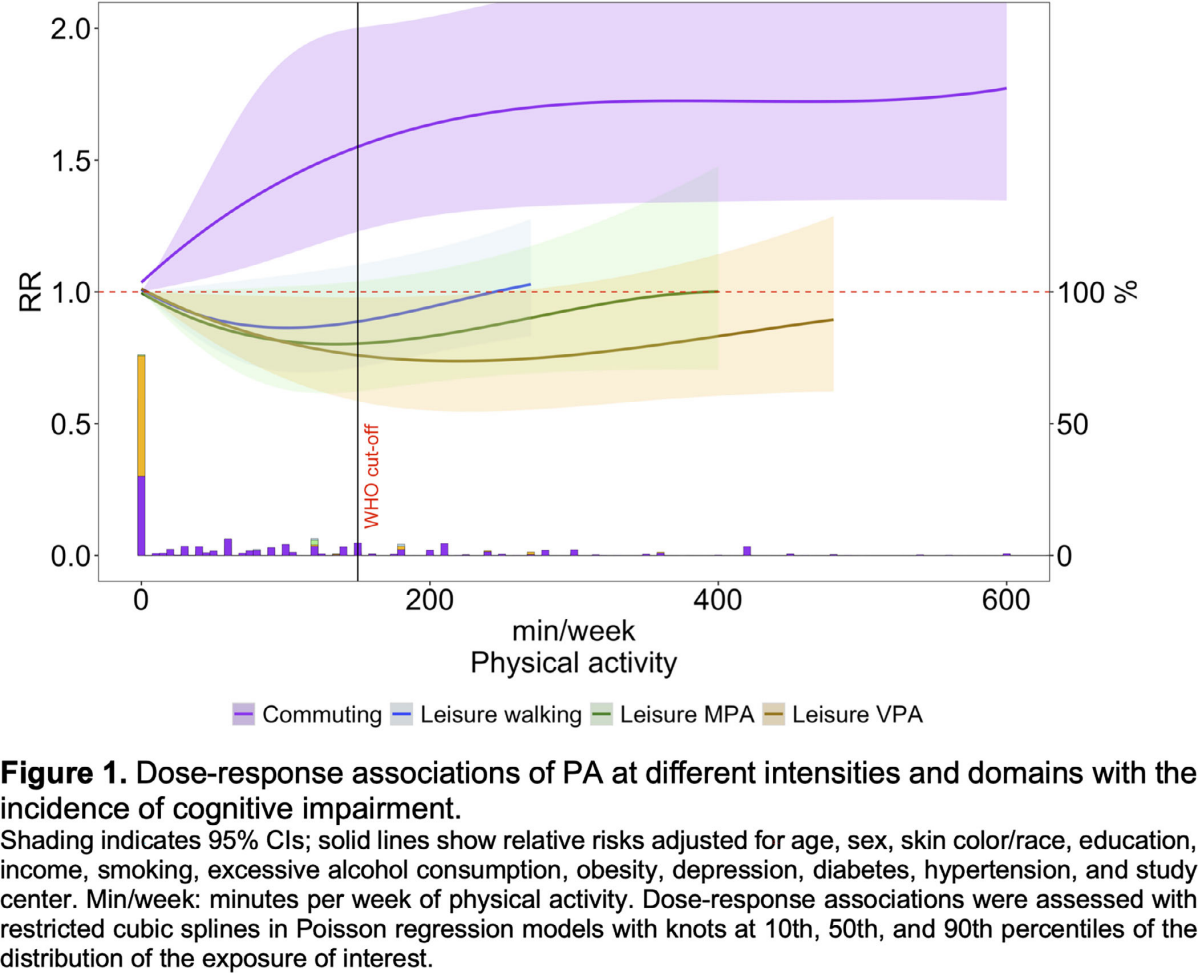# Does “every move count” for cognitive decline? Findings of the ELSA‐Brasil study

**DOI:** 10.1002/alz70860_103809

**Published:** 2025-12-23

**Authors:** Natan Feter, Bruce Duncan, David A Raichlen, Danilo Santos, Jayne Feter, Rodrigo C P Dos Reis, Rosane H Griep, Maria Inês Schmidt

**Affiliations:** ^1^ Universidade Federal do Rio Grande do Sul, Porto Alegre, Brazil; ^2^ University of Southern California, Los Angeles, CA, USA; ^3^ Postgraduate Program in Epidemiology, Universidade Federal do Rio Grande do Sul, Porto Alegre, Rio Grande do Sul, Brazil; ^4^ Universidade Federal do Rio Grande do Sul, Porto Alegre, RS, Brazil; ^5^ Universidade Federal do Rio Grande do Sul, Porto Alegre, Rio Grande do Sul, Brazil; ^6^ Fundação Oswaldo Cruz, Rio de Janeiro, Rio de Janeiro, Brazil; ^7^ Medical School, Universidade Federal do Rio Grande do Sul, Porto Alegre, Rio Grande do Sul, Brazil

## Abstract

**Background:**

Studies investigating the association of physical activity (PA) with cognitive decline usually do not characterize the parameters of PA, especially the domains in life in which PA occurs such as at leisure and commuting. We evaluated the associations of various parameters of PA (volume, frequency, intensity, and context) with cognitive decline in middle‐aged and older adults over an 8‐year follow‐up.

**Method:**

We analyzed data from the *Estudo Longitudinal de Saude do Adulto* (ELSA‐Brasil) study. Participants were enrolled between 2008‐2010, with a follow‐up visit between 2017 and 2019. We used the International Physical Activity Questionnaire – long form to assess PA volume (minutes/week), intensity (light [LPA], moderate [MPA], and vigorous [VPA]), and frequency (1‐2, 3‐4, 5‐6, 7 days/week) in leisure time and commuting contexts at baseline. We evaluated global and domain‐specific (memory, verbal fluency, and executive) cognitive function at baseline and follow‐up. Incident cognitive impairment was defined as a global cognitive function score at follow‐up lower than ‐1.5 SD from the baseline mean. We used robust Poisson regression models to examine the risk of cognitive impairment according to distinct PA paraments. Models were adjusted for sociodemographic, behavioural, and clinical variables. We modelled PA volume as restricted cubic splines due to non‐linear association with the risk of cognitive impairment.

**Result:**

Participants (*N* = 10,187; 57% women) had a mean age of 50.6 (SD: 8.6) years. During a mean follow‐up of 8.1 (SD: 0.6) years, leisure VPA was associated with a reduced risk of cognitive impairment (150 min/week: RR: 0.76; 95%CI: 0.58, 0.98) (Figure 1). Conversely, active commuting (walking or cycling) was associated with an increased risk of cognitive impairment (150 min/week: RR: 1.57; 95%CI: 1.23, 2.00). Leisure MPA was linked to a lower incidence of cognitive impairment (highest significant volume: 67 min/week: RR: 0.83; 95%CI: 0.69, 0.99) while LPA was not linked (*p* ≥0.06) during leisure. PA frequency was not associated with incident cognitive impairment.

**Conclusion:**

The association between PA and the incidence of cognitive impairment depends on PA parameters such as volume, domain, and intensity, especially the latter two. Interventions to promote PA should facilitate opportunities for leisure time PA, especially at higher intensities.